# Creating 12‐lead electrocardiogram waveforms using a three‐lead bedside monitor to ensure appropriate monitoring

**DOI:** 10.1002/joa3.12441

**Published:** 2020-10-05

**Authors:** Kihei Yoneyama, Mayumi Naka, Tomoo Harada, Yoshihiro Akashi

**Affiliations:** ^1^ Division of Cardiology, Department of Internal Medicine St. Marianna University School of Medicine Kawasaki Kanagawa Japan; ^2^ Department of Clinical Laboratory St. Marianna University School of Medicine Hospital Kawasaki Kanagawa Japan

**Keywords:** ECG, Einthoven, diagnoses, arrhythmia, monitor

## Abstract

How do you place the three electrodes to create waveforms for leads I, II, III, aVR, aVL, aVF, and V1‐V6?

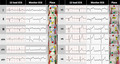

## 1 ELECTROCARDIOGRAM CHALLENGE FOR STUDENT NURSES AND PHYSICIANS

Failure to monitor electrocardiogram (ECG) patterns appropriately can result in adverse events besides incorrect diagnoses. Double‐counting of the heart rate must be avoided. In some cases, it is not possible to place the electrodes on the desired location, especially for surgery on the shoulder or abdomen. After a long monitoring period, it is necessary to replace the tape to protect the patient's skin. Reducing unnecessary alarms makes us become more attentive to real alarms, which helps in the early detection of anomalies and in avoiding noise.

**FIGURE 1:**
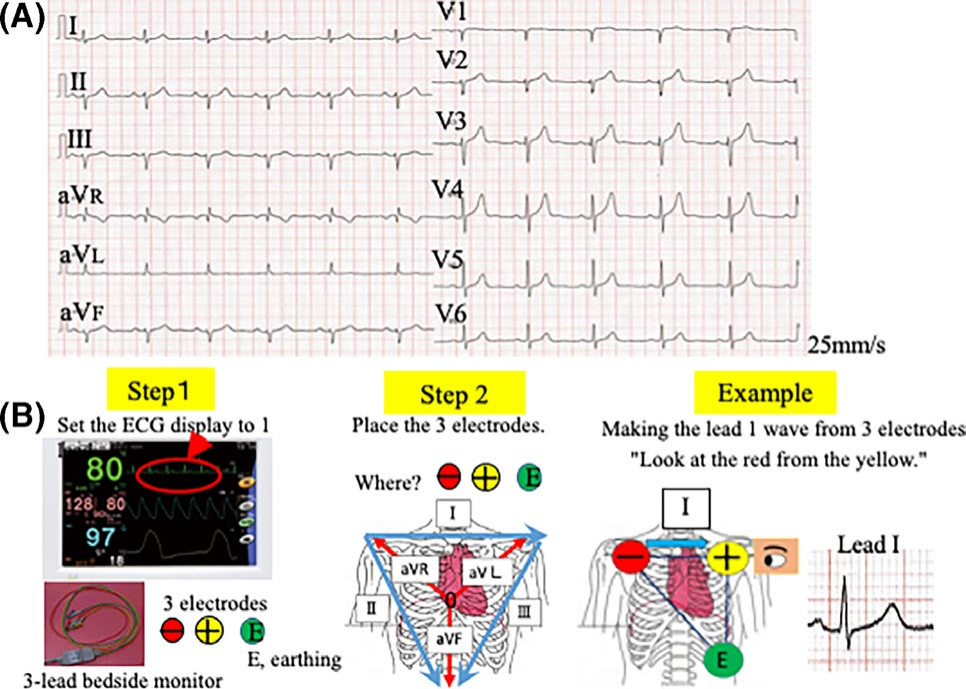
How do you place the three electrodes to create waveforms for leads I, II, III, aVR, aVL, aVF, and V1‐V6? A, The electrocardiogram (ECG) shows left‐axis deviation, suggestive of a left anterior hemiblock. B, Steps required in making 12‐ECG waveforms from a three‐lead ECG. Step 1. Set the ECG display to lead I. Step 2. Place the three electrodes following Einthoven's triangle principle for the limb lead. For example, if you want to make the lead I wave, the three electrodes should be placed as follows: “red to the right shoulder, yellow to the left, and green to the bottom.” Lead I starts from the right hand to the left hand (electrically from negative to positive). In the chest lead, the red electrode is used as a Wilson central terminal

The patient was a 48‐year‐old healthy man (Figure 1A). How do you place the three electrodes to create waveforms for leads I, II, III, aVR, aVL, aVF, and V1‐V6? To address this challenge (Figure 1B), we used Einthoven's triangle principle and then placed the three electrodes, namely red (negative), yellow (positive), and green (earthing).

## 2 DISCUSSION

**FIGURE 2:**
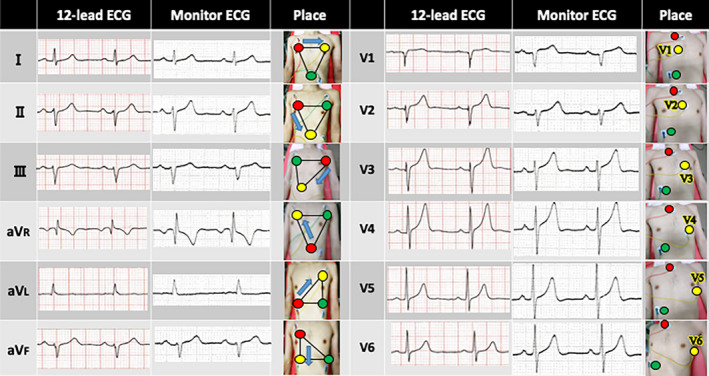
How to create an electrocardiogram (ECG) waveform similar to a standard 12‐lead ECG waveform from a three‐lead bedside monitor. The limb lead is shown in red (negative) and yellow (positive) electrodes. Carefully move the red and yellow electrodes and be aware of Einthoven's triangle principle. The chest lead is also made using the red electrode as a Wilson central terminal. Then, the yellow electrodes were moved at the same location as V1 to V6 on the 12‐lead ECG. The green electrode acts as an earthing and can be placed anywhere without affecting the ECG results for both limb and chest leads

Waveforms similar to that of a 12‐lead ECG can be displayed on the ECG monitor even if only three electrodes are used (Figure 2). In the limb lead, we used Einthoven's triangle principle for leads I‐III and Wilson central terminal for leads aVR, aVL, and aVF.^1^, ^2^ In lead II, the axis goes from the right to the bottom, with the negative and positive electrodes on the shoulder and abdomen, respectively. These positions result in an approximately +60° of orientation, as shown by the blue arrows in Figure 2. In lead III, this axis goes from the left shoulder (negative electrode) to the right or left abdomen (positive electrode). This direction results in approximately +120° of orientation. In lead aVR, where the positive electrode is placed on the right shoulder, you can create the electric axis starting down from the red and yellow electrodes on the right chest. In lead aVL, where the positive electrode is placed on the left shoulder, the direction of the red electrode on the bottom and the yellow on the left chest can be used as the aVL axis. In lead aVF, where the positive electrode is placed on the foot, you can place the red on top and yellow on the bottom to create the aV_F_ axis. This will make a waveform similar to that of lead III.

In the chest lead, the red electrode was used as an “indifferent” electrode, like the Wilson central terminal with 12‐lead ECG.^2^ Chest leads can be generated by fixing the red and moving the yellow electrodes at positions V1 through V6, such as in 12‐lead ECG.

There are three reasons for placing the red electrode on top of the sternum. First, the muscular noise would be less than that on the pectoralis major muscle. Second, the electric potential is greater than that on the pectoralis major muscle. If the red and yellow electrodes are placed in the middle of the sternum, the distance between the red and yellow electrodes becomes small and the electric potential becomes smaller, particularly on the V1 and V2 leads. Third, the QRS (the combination of Q wave, R wave and S wave) waveform will be inverted if the electrodes are placed below the sternum.

The resulting ECG waveform is very similar to the original 12‐ECG waveform, despite possible differences affecting the amplitude. Since this is only a case study, further study is needed in the future.

Data shown in Figure 2 can be used for all cases of three‐electrode monitoring, such as all areas of surgery and hospital units, computed tomography, magnetic resonance imaging, and nuclear medicine requiring ECG synchronization. First, we look at the standard 12‐lead ECG waveform to determine one lead that you want to monitor. Then, we set the ECG monitor to display lead I for the purpose of using this diagram. Then, the electrodes were placed according to that shown in the diagram.

## CONFLICT OF INTEREST

Authors declare no conflict of interests for this article.
